# The Effects of Calcium Phosphate Bone Cement Preparation Parameters on Injectability and Compressive Strength for Minimally Invasive Surgery

**DOI:** 10.3390/bioengineering12080834

**Published:** 2025-07-31

**Authors:** Qinfeng Qiao, Qianbin Zhao, Jinwen Wang, Mingjun Li, Huan Zhou, Lei Yang

**Affiliations:** 1 School of Materials Science and Engineering, Hebei University of Technology, Tianjin 300130, China; qqf190829@163.com (Q.Q.); qbzhao93@163.com (Q.Z.); wangjinwen166@126.com (J.W.); 2 Center for Health Science and Engineering, Hebei Key Laboratory of Biomaterials and Smart Theranostics, School of Health Sciences and Biomedical Engineering, Hebei University of Technology, Tianjin 300131, China; mjli@hebut.edu.cn

**Keywords:** calcium phosphate bone cements (CPCs), microstructure, preparation procedure, injectability, minimally invasive surgery

## Abstract

Compared with biocompatibility, osteoconductivity, and mechanical properties, the poor injectability of calcium phosphate bone cements (CPCs) is always ignored, which actually hinders the development of CPC clinical transfer in minimally invasive orthopedic surgeries. Moreover, currently, CPC preparation in the clinic is labor-intensive and requires well-trained technicists, which might also result in the unstable quality of CPCs. In this work, we focused on three research objectives: (i) introducing a standardized preparation method for CPCs; (ii) studying the effects of preparation parameters on CPC injectability and compressive strength; and (iii) studying the injecting condition effects on CPC injectability, aiming to overcome CPCs’ disadvantages in minimally invasive surgeries. Firstly, two strategies, named “variable mixing barrel control (VMBC)” and the “nested blade–baffle stirring rod (NBBSR)”, were proposed in this study to solve the problems in the preparation of CPCs, which involved blending CPC powder and an agent to generate a paste, by enhancing the mixing performance and mimicking human manual stirring actions. Secondly, although the grinding parameter could significantly generate differences in the microstructure of CPCs, the compressive strength remained relatively stable. However, it was found to significantly affect the injectability of CPCs, leading to the inefficient injection of CPCs. Finally, the effects of syringe design, dimensions, and injecting conditions on CPC injectability were studied, and the results showed that the optimization of these factors enables the injection of CPCs, which has otherwise always been infeasible to implement in minimally invasive orthopedic surgeries.

## 1. Introduction

Bone cement is a commonly used orthopedical clinical material with self-setting and compatible mechanical characteristics [1,2]. Based on these advantages, bone cements are considered to be the best synthetic alternative material for bone grafting treatment in orthopedic and orthodontic applications, remedying the problems of reduced bone mass and deteriorated bone tissue structure [3,4]. In the past few decades, researchers have explored various synthetic and biological materials for bone cements to obtain different functions, such as degradation and osteoconductivity [4,5].

PMMA bone cement was first proposed in 1901 and achieved its landmark clinical application in 1958 based on its stable mechanical characteristics [6]. However, its inability to remodel bone tissue and high stiffness spurred the development of bioactive calcium phosphate cements (CPCs). CPCs began to be used and gradually found applications in orthopedics in the 1980s [7], which was heavily attributed to their bioactivity and features, such as good biocompatibility, osteoconductivity, and biodegradability. However, their complex preparation procedure and low injectability compared with PMMA bone cements were always ignored [8], which actually hindered the implementation of CPCs in minimally invasive orthopedic surgeries, such as vertebroplasty and kyphoplasty [9].

Facing the issue of CPCs’ poor injectability, most researchers have attempted to improve CPC injectability by adding various additives. For example, Ye et al. enhanced the viscosity of CPCs by incorporating modified starch, which has been proven to improve CPC injectability by adjusting the adhesive and swelling properties [10]. Furthermore, Krystyjan et al. added starch with chitosan to successfully develop CPCs with adjustable viscosity according to surgical needs [11]. Meanwhile, Caballero et al. also conducted research combining lysine with CPCs to achieve good injectability [12]. Additionally, our research team found that the use of starch as a binder to prepare CPCs could significantly improve their injectability, which facilitates promising CPC applications in minimally invasive surgeries [13].

Taken together, the injectability of CPCs has obviously improved through various modification methods [14]. However, it should be emphasized that some of these composite bone cements have also induced problems, like high viscosity and tedious preparation procedures [15], which do not meet the requirements of rapid in situ surgical preparation [16]. In our research, it was found that achieving the uniform blending of CPCs would lead to molding challenges in clinical use [17].

On the other hand, Perrot pointed out that during the injection process, particle accumulation could induce liquid phase migration (LPM) [18], which mainly affected the injectability of CPCs. To lessen the occurrence of this phenomenon, besides the modification of compositions, studies have shown that injectability could be adjusted by optimizing external injection conditions [19], such as the design of the syringe, injection conditions, etc. Most recently, Zhang noted that the ease of injection primarily depended on the characteristics of the injection system, including syringe type [20], needle size, injecting speed, etc., based on a computational study by machine learning. For instance, using shorter and wider cannulas and slower injecting speeds could help to improve CPC injectability [21].

In conclusion, it was found that some researchers have noticed problems associated with CPC preparation, such as complex preparation procedures and low injectability. In the initial stage, their research focused on improving injectability by modifying CPC compositions through the addition of additives but always overlooked the problem of complex preparation. Up to now, there have been few reports on the study of the effects of preparation parameters on injectability. Meanwhile, surgeons still complain about the slow and complex preparation procedure of CPCs. In this work, first, the difficulties in CPC preparation were carefully analyzed based on experiments using various types of commercial instruments. To overcome these difficulties, two novel strategies, named “variable mixing barrel control (VMBC)” and the “nested blade–baffle stirring rod (NBBSR)”, were introduced by optimizing instrument design and mimicking well-trained technicists’ manual actions in CPC preparation. Then, their working mechanism was demonstrated by experiments and simulations. The whole blending preparation procedure of CPCs was divided into two steps: (i) mixing of CPC individual components and (ii) cyclic grinding of CPC mixtures. The simulation clearly showed the enhanced mixing performance of the VMBC design without complex structures, and the experiments showed that the preparation eliminates the failure induced by the materials’ high viscosity. At the same time, colorimetric mixing performance test results also proved the feasibility and uniformity of in-barrel mixing of CPCs by our proposed approach with VMBC and the NBBSR. In addition, the CPC microstructures of manual mixing and instrument mixing were captured by SEM to further study the performance of CPC preparation quality. The results of strength testing of well-cured CPCs reflected that the quality of instrument-prepared CPCs was as good as those prepared by well-trained technicists. However, it was found that the grinding preparation parameters of CPCs obviously affected the injectability of CPCs. Last but not least, the effects influencing CPC injectability were also comprehensively discussed in this work, including the design and dimensions of the syringe, injecting speed, and testing ambient temperature. Overall, this work proposed the potential surgical instrument design for CPC injection applications.

## 2. Materials and Methods

### 2.1. Materials and Preparation of CPC

Analytical dicalcium phosphate dihydrate (DCPD; Sigma Aldrich, St. Louis, MO, USA), sodium hydrogen phosphate (Na_2_HPO_4_, Sigma Aldrich), barium sulfate (BaSO_4_, Sigma Aldrich), and α-tricalcium phosphate (α-TCP, Dingan Science and Technology, Suzhou, China) were ball-milled in ethanol. The base materials of the CPC were prepared according to the preparation protocol reported in our previous study, which contained α-TCP and DCPD at a mass ratio of 9:1 [22]. CPC powder was prepared by mixing CPC base materials, pregelatinized starch, and BaSO_4_ powder at a weight ratio of 3:1:1; the setting liquid was 0.25 mol/L Na_2_HPO_4_ solution [23]. The CPC was finally prepared with a powder-to-liquid ratio of 2.5 [24].

### 2.2. Rheological Measurement

The rheological properties of the CPC were studied using a rheometer (AR2000, TA Instruments, New Castle, DE, USA) equipped with a temperature control unit in oscillating mode with a plate–plate configuration (plate diameter, 20 mm; truncation gap, 1000 µm). Sweep tests were conducted from 0.1 to 10 rad/s. The temperature was set to 25 °C, and the oscillating strain was 1%.

### 2.3. Mechanical Property Test

The cement paste was filled into a cylindrical mold (6 mm in diameter and 12 mm in height) at room temperature (~25 °C) for 10 min. The semi-solid cement was then pushed out from the mold and kept in an incubator (DHTH-27-20-P-SD, Doaho Machinery Co., Ltd., Shanghai, China) at 37 °C and 95% relative humidity (RH) for 3 days. Cylindrical cement samples were uniformly polished by 1200-grade sandpaper to ensure parallel and smooth top and bottom surfaces. The compressive strength of the cement was tested through a universal mechanical tester (HY-1080, HengYi Precision Instrument Co., Ltd., Shanghai, China) with a 5 kN load cell at a cross-head speed of 1 mm/min, as reported previously [25]. The measurement of all samples was repeated at least five times.

### 2.4. Scanning Electron Microscopy (SEM)

The morphology of the cross-sections of the bone cement was captured by SEM (JSM-7100 F, JEOL Ltd., Tokyo, Japan). After setting for 3 days, the cylindrical cement samples were snapped to expose the cross-section. Images were taken at an accelerating voltage of 15 kV. All of the samples were pre-coated with Au-Pd coating (SC7620, Quorum Technologies, Lewes, East Sussex, UK) before the test.

### 2.5. Injectability of the Bone Cement

The injectability of bone cement was evaluated using a standardized protocol. The cement paste was loaded into customized syringes and extruded at a constant speed of 10 mm/min via a universal testing machine (HY-1080, HengYi Precision Instrument Co., Ltd., Shanghai, China). Force monitoring commenced upon plunger–syringe contact, with the injecting force continuously recorded throughout extrusion. The steady extrusion phase (excluding initial peak force and unloaded terminal segment) was quantitatively analyzed, and the mean injecting force during this stable period was calculated as the key metric for injectability assessment.

### 2.6. Setting Time

The setting time of the bone cement was measured according to ASTM C191-03. The freshly prepared cement paste was packed into a mold (6 mm in diameter and 12 mm in height) and flattened with a spatula. A Vicat device with an initial needle (113.4 g in weight and 2.12 mm in diameter) and a final needle (453.6 g in weight and 1.06 mm in diameter) was used to test the initial setting time and final setting time, respectively. Each sample was tested five times, and the average value was calculated.

### 2.7. Collapsibility in Water

The collapsibility of the cement paste in water was tested by injecting freshly prepared paste into deionized water using a 5 mL syringe. The cement extrudate was shaken on a shaker (SK-0330-Pro, Scilogex, Rocky Hill, CT, USA) at 180 r/min for 1 min and then left in deionized water at 37 °C for 24 h. If the bone cement did not collapse, it indicated that it had good resistance to collapse [13].

### 2.8. RGB Colorimetry

RGB colorimetric methods were conducted using ImageJ 1.53j (National Institutes of Health, Bethesda, MD, USA). In the study of image analysis of the CPC with color ink, the sample state was first recorded using the camera. Then, the captured image files were opened using the image processing software ImageJ, and two representative lines were selected from them for comparative analysis. The images were converted from RGB color mode to individual color channels to analyze the color intensity of the testing points.

### 2.9. Design and Fabrication Method

The prototype device was initially designed using AutoCAD 2020 (FAutodesk, San Rafael, CA, USA) software, and the fabrication employed both FDM (Fused Deposition Modeling) and SLA (Stereolithography) 3D printing technologies. The main structural components were fabricated using industrial-grade FDM 3D printing with PLA material, with a printing precision of 0.1 mm. For the stirring rod, which required higher surface friction and structural strength, SLA 3D printing was utilized with a stainless steel material.

### 2.10. Statistical Analysis

Statistical analysis was performed using Origin software (Version OriginPro 2024b, Learning Edition, Northampton, MA, USA). All experiments were replicated at least three times to ensure data reliability, and the data were expressed as mean ± standard deviation (SD). Prior to statistical comparisons, the normality of all datasets was confirmed using the Shapiro–Wilk test. For multiple comparisons, one-way analysis of variance (ANOVA), followed by Tukey’s post hoc test, was applied, and statistical significance was defined as *p* < 0.05.

## 3. Results and Discussion

### 3.1. CPC Preparation Procedure and Difficulties

**Figure 1:**
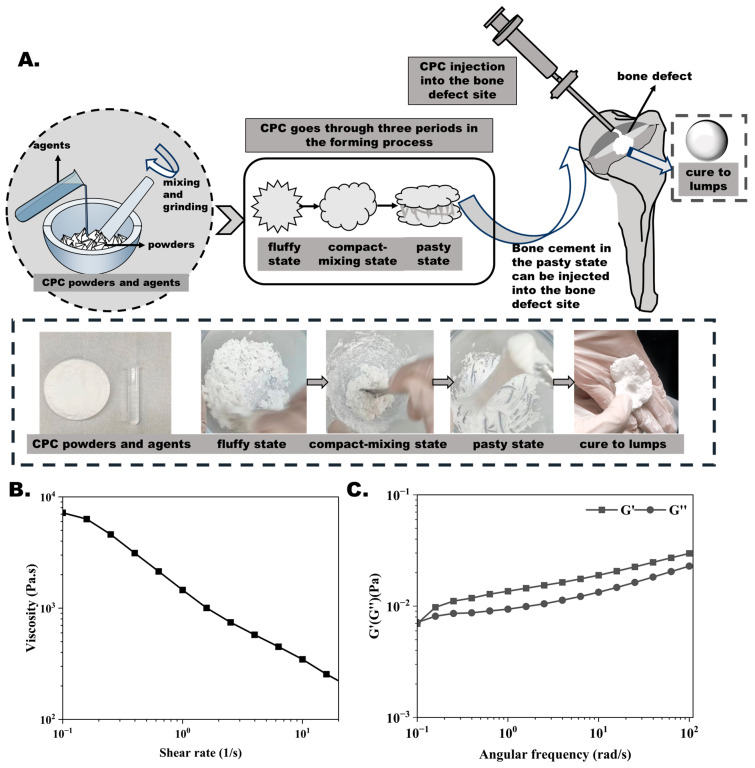
CPC forming process and rheological behavior: (**A**) CPC preparation process with four stages of molding. (**B**) CPC viscosity with shear rate. (**C**) Energy storage modulus (G′) and loss modulus (G″) of CPCs.

As shown in Figure 1A, in the preparation scheme of CPCs, CPC powders and agents experience three transitions with different physical states, including the fluffy state, the compact-mixing state, and the pasty state, and, finally, cure to lumps. In minimally invasive surgery, CPC pastes would normally be prepared by a well-trained technicist in an open vessel using a stirring rod for 5 to 10 min [26]. Then, the bone cement in the pasty state could be transferred into a syringe and injected into the bone defects. In Figure 1B,C, the rheological results demonstrated that the CPCs exhibited typical pseudoplastic fluid characteristics, with the shear thinning phenomenon.

However, for PMMA bone cements, which have been widely adopted in minimally invasive surgery, the preparation procedure merely requires less than 1 min. PMMA powders and agents can be rapidly and well prepared at different ratios, depending on the surgery requirements, in a customized syringe barrel and then injected directly. This is one of the most important reasons for the popularity of PMMA bone cements. On the contrary, CPCs must be prepared by a well-trained technicist in an open vessel, and the process requires more than 5 min [27]. As a result, preparing CPCs in an open vessel might induce contamination [5], and the transfer of CPCs from the vessel to the syringe could increase the labor burden of surgeons. On the contrary, PMMA bone cements could be prepared quickly and easily in a syringe barrel and then injected directly. Consequently, the similar usage schemes and methods of CPCs and PMMA bone cements have been the necessary application direction for minimally invasive surgical bone cements.

**Figure 2:**
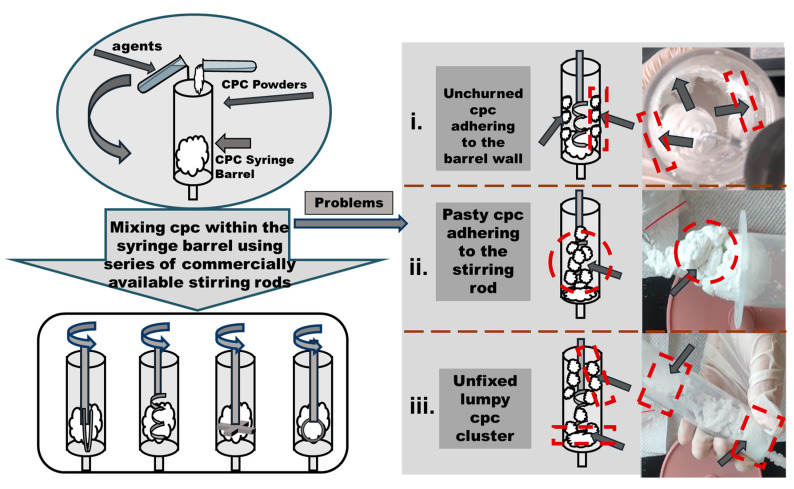
CPC preparation in a syringe barrel (similar to PMMA bone cements). Three typical phenomena of failed CPC preparation in testing: (**i**) unchurned CPC adhering to the barrel wall; (**ii**) pasty CPC adhering to the stirring rod; (**iii**) unfixed lumpy CPC clusters. The dotted-line boxes and arrows depict the uncontrollable material clusters in the barrel.

By testing the mixing of CPCs within the syringe barrel using a series of commercially available stirring rods, it was found that the formation of CPCs was impossible because of the high viscosity of the CPC mixture, as shown in Figure 2. There were three typical problems and phenomena commonly observed using commercial stirring rods, categorized as follows: (i) unchurned CPC adhering to the barrel wall; (ii) pasty CPC adhering to the stirring rod; and (iii) unfixed lumpy CPC clusters. Because of these issues, no relevant instrument could be adapted to CPC preparation so far.

### 3.2. Strategies of VMBC and NBBSR

The manual CPC preparation in the open vessel was followed by a certain protocol, in which the CPC powder was first mixed with the agent uniformly and then ground in a blender by repeated pressing. As a result, to overcome the challenges of CPC preparation within the barrel, the above-mentioned failed phenomena were expected to be eliminated through the imitation of manual blending operations.

**Figure 3:**
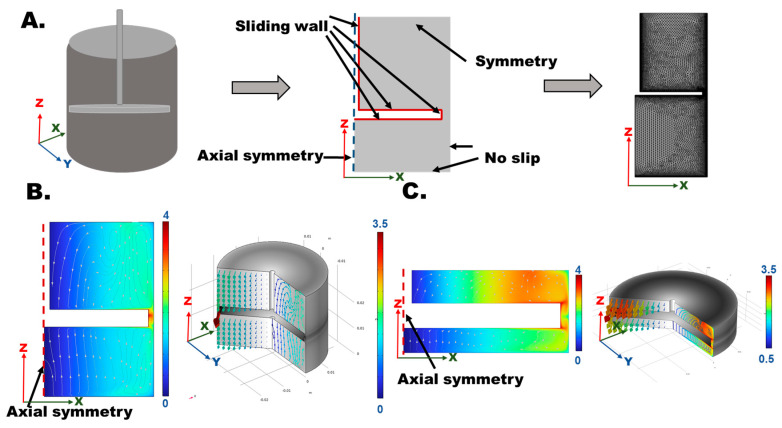
Simulation of the VMBC design for CPC mixing. (**A**) Validation of the VBMC Comsol simulation mechanism was conducted to analyze the effects using a built 2D syringe barrel and stirring rod model. The setting boundary condition and mesh are also shown here. (**B**) The secondary flow vortex and rotating vortex field in the cross-section of the barrel with a stirring rod at the high-volume VBMC condition. The streamline plot shows the combination of fluid flow velocity in the x and z directions. The background contour plot is associated with the pressure distribution, where the unit is Pascals (Pa). The right plot shows the rotating vortex in the three-dimensional simulation model. (**C**) The secondary flow vortex and rotating vortex field in the cross-section of the barrel with a stirring rod at the low-volume VBMC condition. The streamline is the combination of the fluid flow velocity in the x and z directions. The background contour is associated with the pressure distribution, and the unit is Pascals (Pa). The right plot shows the rotating vortex in the three-dimensional simulation model.

First, for the mixing of CPC individual components, in response to the volume change between different CPC stages, a “variable mixing barrel control” (VMBC) design was proposed to adjust the barrel space following the CPC stage and allow our syringe to improve blending performance at the same time. As shown in Figure 3A, a two-dimensional barrel with a stirring rod model was built based on rotational symmetry features. The walls’ boundary conditions were set as no slip and rotating, and the angular velocity of the mixing bar (sliding wall) was 0.785 rad/s (0.25π rad/s). In Figure 3B,C, the rotating vortex intensity in the y direction and secondary flow vortex in the x-z plane are depicted by the arrow and streamline plots in the simulation results. The computation results showed that the vortex intensity caused by rotating the rod around the blade (rotating vortex and secondary flow vortex) increased significantly when the barrel space gradually decreased at the same rotating condition (Figure 3B,C). This was expected to enhance the mixing motion intensity between the powders and agents, which could solve the problem induced by the CPC’s high viscosity.

**Figure 4:**
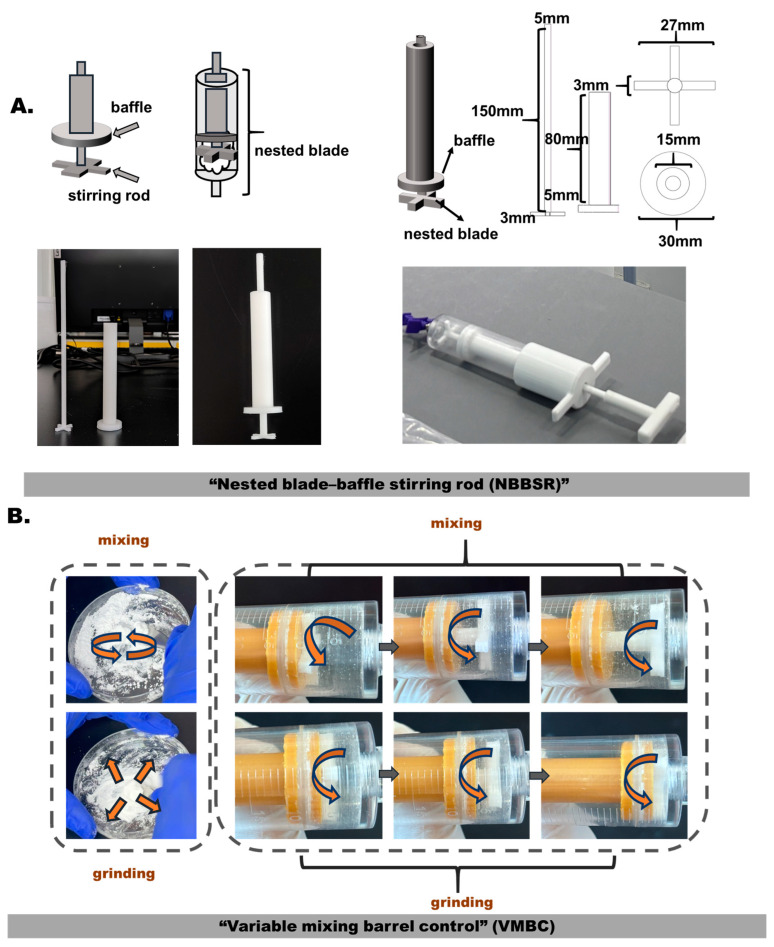
VMBC and NBBSR designs and the fabricated instrument. (**A**) Schematics and a real photograph of the proposed instrument based on the VMBC and NBBSR designs. (**B**) Manual preparation of CPCs by well-trained technicists included two main steps in the whole procedure: uniform mixing and cyclic grinding. The right images show the mimicking mixing and grinding actions using the proposed instrument. The direction of the arrow represents the direction in which the cement is mixed and ground by the tool.

Then, for the cyclic grinding of the CPC mixture, a biomimetic nested stirring rod was introduced to replicate the manual operations in the barrel space. A shown in Figure 4A, a “nested blade–baffle stirring rod (NBBSR)” was proposed to enable the replication of the manual cyclic grinding actions of skillful technicists within the barrel space. The NBBSR consisted of two main parts: a rotating blade and a baffle with a seal ring. The two parts formed a nested assembly that ensured the independent movement of each part. At the same time, the designed NBBSR could implement the VMBC method by its baffle. As shown in Figure 4A, the prototype instrument with the NBBSR and VMBC was fabricated using 3D printing technology. It was coupled with a 50 mL barrel to test the CPC preparation.

As shown in Figure 4B, firstly, the NBBSR could scratch the barrel wall with the baffle component, which enabled all of the CPC powder and agent to be mixed within the barrel, avoiding CPC adherence to the barrel wall. Otherwise, the combined use of the blade rod and the baffle rod was capable of mimicking the grinding action through the mutual friction, which prevented the pasty CPC from adhering to the stirring rod and unfixed lumpy CPC clusters within the barrel. Additionally, the movable baffle with rubber seal ring could decrease the mixing space as the CPC stage changed to conduct the VMBC method. Finally, the results showed that the prototype instrument was able to complete the preparation of CPCs within the syringe barrel in 5 min (Figure 4B).

### 3.3. Study of CPC Mixing Quality Using VMBC and the NBBSR

**Figure 5:**
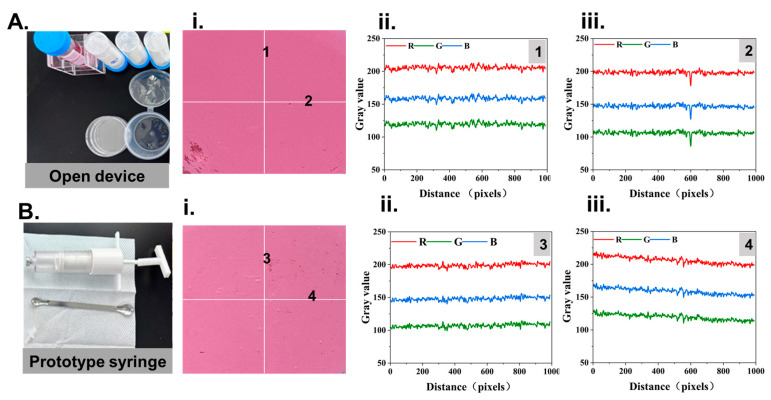
Study of CPC mixing quality between skillful technicists. (**A**) CPCs prepared by skillful technicists. (**i**) Sampling lines 1 and 2 of CPCs. (**ii**) The analyzed RGB intensity of points on sampling line 1. (**iii**) The analyzed RGB intensity of points on sampling line 2. (**B**) CPCs prepared by our proposed prototype instrument. (**i**) Sampling lines 3 and 4 of CPCs. (**ii**) The analyzed RGB intensity of points on sampling line 3. (**iii**) The analyzed RGB intensity of points on sampling line 4.

Then, the mixing efficacy between manual operation by a skilled technicist using an open device and our prototype instrument was quantitatively evaluated using 5 g of the calcium phosphate cement (CPC) with 2 mL of an aqueous colorant. As shown in Figure 5, the mixing quality was evaluated through colorimetry. By adding a certain amount of aqueous color ink into the agent of the CPC, colored CPC pastes were obtained after mixing by both methods. In this testing, the RGB color intensity analysis at every point on the sampling lines in the pastes reflected the consistency of color, which represented the mixing performance. The RGB intensity plots in Figure 5 show that the tested RGB intensity of points on each sampling line maintained good stability. And the average intensity between each sampling line was nearly close to R:201, G:111, and B:151.3, demonstrating that the mixing performance of the two methods was nearly the same process.

### 3.4. Grinding Effects

**Figure 6:**
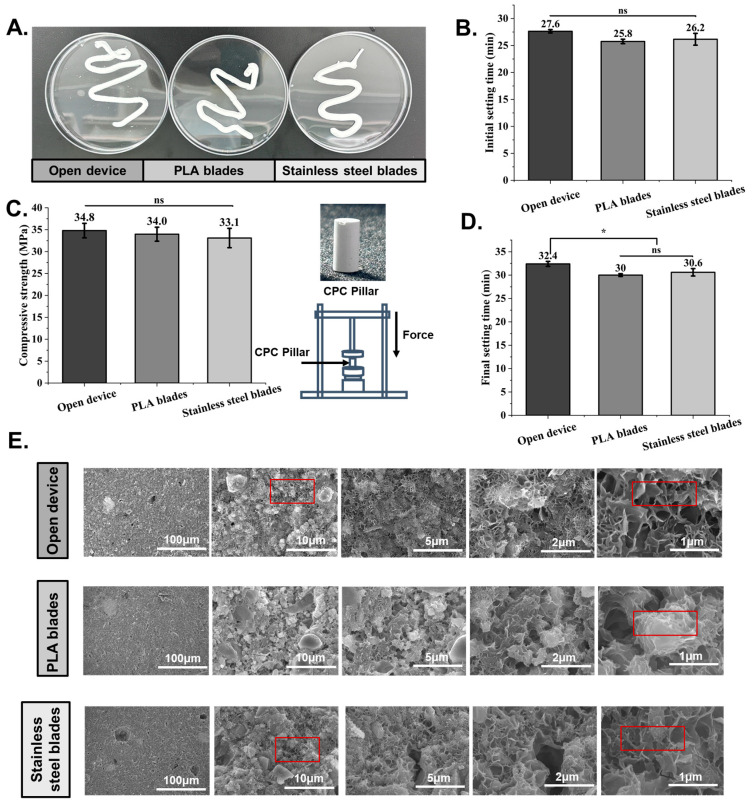
Effects of grinding performance on CPC mechanical properties. (**A**) Anti-collapsibility of CPCs in deionized water. (**B**) Initial setting time tests. (**C**) Final setting time tests. (**D**) Compressive strength tests (ns, no statistical difference; *, *p* < 0.05). (**E**) SEM micrographs of CPC cross-sections after setting for 3 days. The picture frames highlight the large particles and voids that are created when the bone cement is not adequately mixed.

The collapsibility in water and the compressive strength of the CPC pastes prepared using the different methods were also tested. Here, we tested three conditions, including manual grinding using a metal stirring rod, instrument grinding using the NBBSR with PLA blades, and instrument grinding using the NBBSR with metal blades, which could provide stronger grinding effects. As shown in Figure 6, the prepared CPCs showed good anti-collapsibility in water. Meanwhile, as shown in Figure 6B,D, the results of the curing time of the CPCs prepared by the different methods indicated that the CPCs prepared by the technicist had a longer curing time, whereas the CPCs prepared by the prototype instrument had a shorter curing time. This might be due to the closed environment of the barrel and heat induced by the mutual friction of NBBS, which accelerated the curing of CPCs. Also, it was found that the metal blades were able to transfer heat faster because of their good thermal conductivity, resulting in a shorter bone cement curing time.

Furthermore, as shown in Figure 6C, the compressive strength of CPC anti-collapsibility was tested to investigate the quality of the CPCs based on their mechanical properties. It was predicted that the grinding performance of the CPCs in the preparation procedure would influence the properties after forming. However, it was shown that the compressive strength of anti-collapsibility was nearly the same among the three tests.

Moreover, although factors, including the compressive strength, did not change significantly with the different effects of grinding, the SEM cross-section images of the CPC pastes in Figure 6E show that a tidier network microstructure with uniform particles was found in the pastes prepared by the technicist. On the contrary, for the NBBSR with PLA blades, the presence of incompletely ground large-size particles was found at different magnifications. Importantly, these large and uneven dimension particles were rarely observed when the blade material was stainless steel. These results indicated that stronger grinding and crushing effects contribute to the smaller milled CPC particles and finer solidified microstructure of CPC pastes. Although stronger grinding effects were not the critical factor in the cured CPC pastes’ mechanical properties, it was expected that they would significantly influence the injectability of CPC pastes based on the LPM mechanism in references [28].

### 3.5. Effects of Injecting Conditions on CPC Injectability

**Figure 7:**
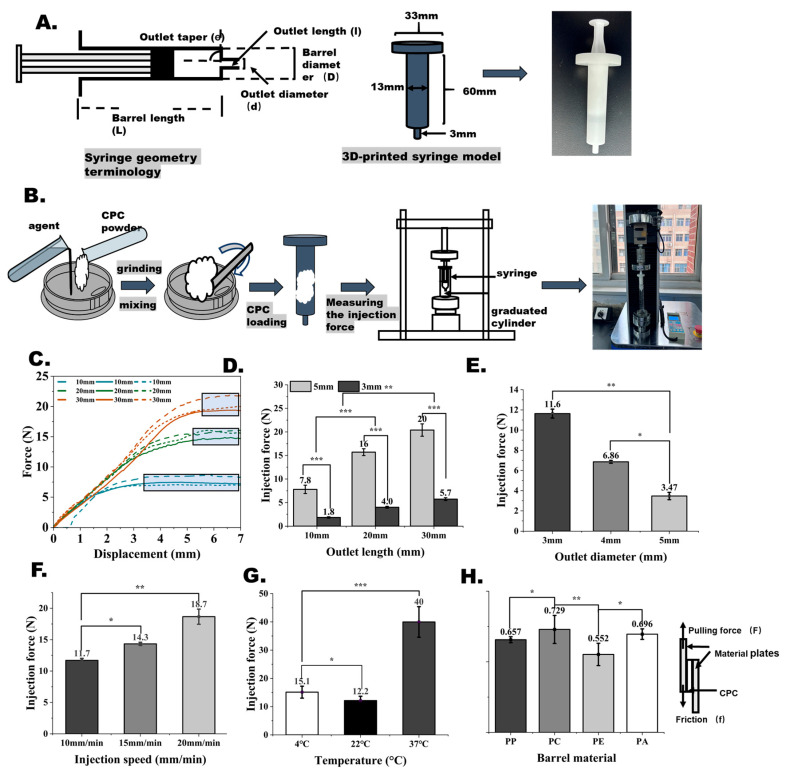
Effects of objective conditions on CPC injectability. (**A**) Customized syringe used in the tests. (**B**) Test scheme of CPC injectability. (**C**) The plot of the injecting force recorded in the process of the injecting test. (The box indicates the value of the injecting force taken by the cement for smooth injection.) (**D**) Effects of the syringe outlet length on CPC injectability. (**E**) Effects of the syringe outlet diameter on CPC injectability. (**F**) Effects of the injecting speed on CPC injectability. (**G**) Effects of ambient temperature on CPC injectability. (**H**) Effects of material on CPC injectability (ns, no statistical difference; *, *p* < 0.05; **, *p* < 0.01; ***, *p* < 0.001).

Moreover, the injectability of the CPCs was also studied comprehensively. In this work, instead of additive effects, we focused on the effects of the instrument and operating environment, including barrel dimensions, injecting speed, instrument material, and ambient temperature [29]. First, to test the effects of the syringe design and dimensions, customized syringes with different structural designs and dimensions were designed in this study and fabricated using 3D printing to conduct injectability testing (Figure 7A). The full test scheme is shown in Figure 7B. The prepared CPC pastes were loaded into the customized syringe barrel and injected through a mechanical testing machine to record the injecting force in the process of CPC injection, as shown in Figure 7C. The value of the injecting force was calculated as the mean of all the achieved forces over 95% of the recorded highest injecting forces in each measurement.

In Figure 7D, the results show that the force gradually increased with increasing outlet length, which was attributed to the increased outlet resistance. Meanwhile, in Figure 7E, it is clearly shown that the force gradually decreased as the outlet diameter increased from 3 to 5 mm, which was attributed to the weakening of the outlet resistance. Next, as shown in Figure 7F, we tested the effects of injecting speed on the injectability of CPC pastes and found that increasing the injecting speed led to a rise in the injecting force, which was totally opposite to the perspectives on CPCs given by other researchers previously. This phenomenon might be attributed to the viscoelastic characteristics of CPC paste [30]. In addition, as shown in Figure 7G, experiments were conducted at different temperature conditions of 4 °C, 22 °C, and 37 °C to evaluate the effects of temperature on injectability. The results showed that the injecting forces at 4 °C and room temperature (22 °C) were nearly the same, while the highest injecting force was required at 37 °C. This was because the high temperature would accelerate the curing of CPC pastes and lower the mobility of CPC pastes [31].

Additionally, we found that CPCs suffer different friction and adhesion effects on different materials. In Figure 7H, four kinds of materials, including PA, PP, PE, and PC, were used to study the effects of different materials on injectability through tensile experiments that simulated the extrusion process of CPC pastes in the barrel. The results showed that PE induced the lowest friction shear force, around 30% lower than the others. In summary, the series of experimental results showed that the syringe outlet length, diameter, barrel material, injecting speed, and operating temperature all affect CPC paste injectability. These rigorous experimental results are expected to be the cornerstone of the guide for CPC instrument design and relevant clinical operations.

### 3.6. Effects of Grinding Performance on CPC Injectability

**Figure 8:**
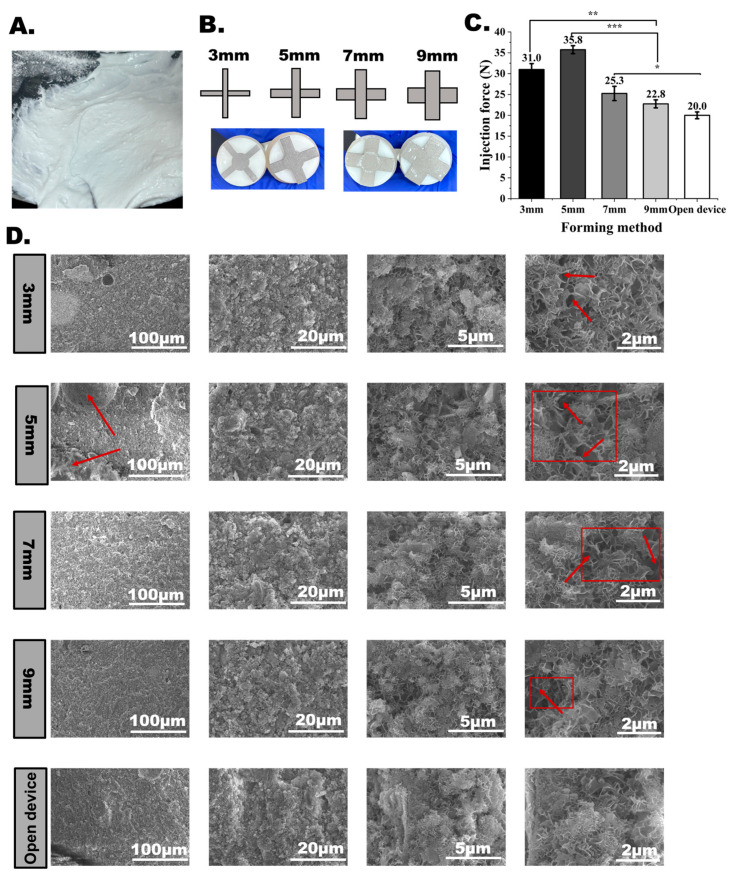
Effects of grinding performance on CPC injectability. (**A**) Wrapped solid microparticles in CPC pastes prepared by the PLA blade. (**B**) Blades with different widths. (**C**) Effects of blade width on CPC injectability (ns, no statistical difference; *, *p* < 0.05; **, *p* < 0.01; ***, *p* < 0.001). (**D**) SEM micrographs of CPC cross-sections prepared using different wide blades and open vessels. The picture frames highlight the large particles and voids that are created when the bone cement is not adequately mixed.

In the CPC preparation study, the stronger grinding effects of the stainless steel blade could produce a finer paste microstructure. As shown in Figure 8A, for the paste without enough cyclic grinding treatment, a few visible solid CPC particles still could be observed wrapped within the pastes, which might be identified as a failure product by surgeons in clinical practice. Although the finer microstructure of the CPCs did not obviously affect the mechanical properties of the cured CPC pastes, it was expected that the finer microstructure of CPCs with smaller microparticles could generate better injectability than the coarse microstructure with large microparticles [17]. This could play an important role in the injectability of CPCs. In our experiments shown in Figure 8B, different grinding effects could be generated by increasing the blade dimensions, and the width of the blades was increased from 3 to 9 mm. It was shown that the injectability of the CPC paste ground by the larger blades tended to achieve better injectability with a lower injecting force, as shown in Figure 8C. Although the CPC pastes prepared by the technicist in the open vessel still possessed the best injectability, the injectability of the CPC pastes prepared using the 9 mm wide blades was almost the same. Moreover, as shown in Figure 8D, the SEM images of the different samples confirmed that the wider stainless steel blade could generate a finer and denser CPC microstructure.

## 4. Conclusions

In this work, we focused on two critical problems that impeded the clinical transfer of CPCs for minimally invasive orthopedic surgeries to replace PMMA bone cements. Another aim of this work was to provide a potential surgical instrument design for CPCs in minimally invasive orthopedic surgeries so that they can be used in a similar protocol to PMMA bone cements. The complicated CPC preparation procedure and poor operating experience have glossed over their material advantages and hindered the development of CPCs. The preparation parameters and injectability were quantitatively studied. To address the challenges of preparing CPCs, two innovative strategies, named VMBC and the NBBSR, enabled the in-barrel preparation in ~ 5 min by mimicking the manual actions of mixing and grinding. The simulation and experiment results showed that the dynamic volume change in the barrel by VMBC could significantly improve the mixing intensity with the phase change in CPCs. On the other hand, the NBBSR could minimize the dead volume of CPC preparation and mimic human manual actions to enable the CPC blending preparation in a syringe barrel. The results showed that the mechanical properties prepared by the proposed protocol were nearly the same as the CPCs prepared by a skillful technicist. On the other hand, different from the popular research interests in injectability through additives, the effects of instrument and operating conditions on CPC injectability were tested and discussed. It was shown that the injectability of CPCs could be optimized by adjusting the instrument design and operating environment, paving the way for their clinical application in minimally invasive orthopedic surgeries. Finally, it was clearly demonstrated that the blending preparation of CPCs would directly influence their injectability. Overall, the improvement in CPCs for clinical transfer application in minimally invasive surgeries should be comprehensively considered among the factors of preparation parameters, compositions, and injection conditions.

## Data Availability

All data generated or analyzed during this study are included in this published article.

## References

[1] Liu W., Li Y., Zhou C., Yu T. (2024). A ternary calcium silicate bone cement with high mechanical properties and good operability. Ceram. Int..

[2] Gamal S., Mikhail M., Salem N., EL-Wakad M.T., Abdelbaset R. (2025). Enhanced bone cement for fixation of prosthetic joint utilizing nanoparticles. J. Mater. Sci. Mater. Med..

[3] Auran R., Movassaghi K., Nam D., Heckmann N. (2024). Bone cement in adult hip and knee reconstruction: A review of commercially available options and clinical outcomes. J. Am. Acad. Orthop. Surg..

[4] Arkin V.H., Narendrakumar U., Madhyastha H., Manjubala I. (2021). Characterization and in vitro evaluations of injectable calcium phosphate cement doped with magnesium and strontium. ACS Omega.

[5] Belaid H., Barou C., Collart-Dutilleul P.-Y., Desoutter A., Kajdan M., Bernex F., Tétreau R., Cuisinier F., Barés J., Huon V. (2022). Fabrication of radio-opaque and macroporous injectable calcium phosphate cement. ACS Appl. Bio. Mater..

[6] Galibert P., Deramond H., Rosat P., Le Gars D. (1987). Preliminary note on the treatment of vertebral angioma by percutaneous acrylic vertebroplasty. Neurochirurgie.

[7] Kuehn K.-D., Ege W., Gopp U. (2005). Acrylic bone cements: Composition and properties. Orthop. Clin. N. Am..

[8] Lu Q., Liu C., Wang D., Liu H., Yang H., Yang L. (2019). Biomechanical evaluation of calcium phosphate-based nanocomposite versus polymethylmethacrylate cement for percutaneous kyphoplasty. Spine J..

[9] Chen S., Yang D., Zhuo C., Zhou Z., Aleem H.B., Huang L., Chen H. (2024). Comparative analysis of percutaneous vertebroplasty and kyphoplasty in the treatment of Stage III Kummell’s disease without neurological symptoms: A retrospective study. J. Orthop. Surg. Res..

[10] Verron E., Pissonnier M.-L., Lesoeur J., Schnitzler V., Fellah B.H., Pascal-Moussellard H., Pilet P., Gauthier O., Bouler J.-M. (2014). Vertebroplasty using bisphosphonate-loaded calcium phosphate cement in a standardized vertebral body bone defect in an osteoporotic sheep model. Acta Biomater..

[11] Krystyjan M., Khachatryan G., Grabacka M., Krzan M., Witczak M., Grzyb J., Woszczak L. (2021). Physicochemical, bacteriostatic, and biological properties of starch/chitosan polymer composites modified by graphene oxide, designed as new bionanomaterials. Polymers.

[12] Wang X.-H., Jia S.-J., Hao D.-J. (2020). Advances in the modification of injectable calcium-phosphate-based bone cements for clinical application. Chin. Med. J..

[13] Zhang R., Liu H., Zhou H., Liang C., Wang X., Yang H., Bai Y., Yang L. (2022). Pregelatinized starch as a cohesion promoter improves mechanical property and surgical performance of calcium phosphate bone cement: The effect of starch type. Mater. Technol..

[14] Ribeiro N., Reis M., Figueiredo L., Pimenta A., Santos L.F., Branco A.C., de Matos A.A., Salema-Oom M., Almeida A., Pereira M. (2022). Improvement of a commercial calcium phosphate bone cement by means of drug delivery and increased injectability. Ceram. Int..

[15] Guan J., Liu L., Li L., Xie C., Khan M. (2024). Mesoscopic model for the fracture of polymethyl methacrylate bone cement. Eng. Fract. Mech..

[16] Ruan S., Deng J., Yan L., Huang W. (2018). Evaluation of the effects of the combination of BMP-2-modified BMSCs and PRP on cartilage defects. Exp. Ther. Med..

[17] Zhang J.T., Tancret F., Bouler J.M. (2011). Fabrication and mechanical properties of calcium phosphate cements (CPC) for bone substitution. Mater. Sci. Eng. C.

[18] Perrot A., Lanos C., Melinge Y., Estellé P. (2007). Mortar physical properties evolution in extrusion flow. Rheol. Acta.

[19] Sui P., Yu T., Sun S., Chao B., Qin C., Wang J., Wang E., Zheng C. (2023). Advances in materials used for minimally invasive treatment of vertebral compression fractures. Front. Bioeng. Biotechnol..

[20] Bayfield M., Haggett J.A., Williamson M.J., Zargar A., Wilson D.I. (1998). Liquid phase migration in the extrusion of icing sugar pastes. Food Bioprod. Process.

[21] Xu H.H., Wang P., Wang L., Bao C., Chen Q., Weir M.D., Chow L.C., Zhao L., Zhou X., Reynolds M.A. (2017). Calcium phosphate cements for bone engineering and their biological properties. Bone Res..

[22] Liu H., Zhang Z., Gao C., Bai Y., Liu B., Wang W., Ma Y., Saijilafu, Yang H., Li Y. (2020). Enhancing effects of radiopaque agent BaSO4 on mechanical and biocompatibility properties of injectable calcium phosphate composite cement. Mater. Sci. Eng. C.

[23] Liu H., Guan Y., Wei D., Gao C., Yang H., Yang L. (2016). Reinforcement of injectable calcium phosphate cement by gelatinized starches. J. Biomed. Mater. Res. B Appl. Biomater..

[24] Sun H., Liu C., Liu H., Bai Y., Zhang Z., Li X., Li C., Yang H., Yang L. (2017). A novel injectable calcium phosphate-based nanocomposite for the augmentation of cannulated pedicle-screw fixation. Int. J. Nanomedicine.

[25] Richter R.F., Vater C., Korn M., Ahlfeld T., Rauner M., Pradel W., Stadlinger B., Gelinsky M., Lode A., Korn P. (2023). Treatment of critical bone defects using calcium phosphate cement and mesoporous bioactive glass providing spatiotemporal drug delivery. Bioact. Mater..

[26] Zhou H., Liang C., Wei Z., Bai Y., Bhaduri S.B., Webster T.J., Bian L., Yang L. (2019). Injectable biomaterials for translational medicine. Mater. Today.

[27] Moussi H., Weiss P., Le Bideau J., Gautier H., Charbonnier B. (2022). Injectable macromolecule-based calcium phosphate bone substitutes. Mater. Adv..

[28] Rabideau B.D., Moucheront P., Bertrand F., Rodts S., Mélinge Y., Lanos C., Coussot P., Franks G. (2012). Internal flow characteristics of a plastic kaolin suspension during extrusion. J. Am. Ceram. Soc..

[29] Peng Y.M., Unluer C., Shi J.Y. (2021). Rheo-viscoelastic behavior and viscosity prediction of calcium sulphoaluminate modified Portland cement pastes. Powder Technol..

[30] Libos I.L.S., Cui L., Liu X. (2021). Effect of curing temperature on time-dependent shear behavior and properties of polypropylene fiber-reinforced cemented paste backfill. Constr. Build. Mater..

[31] Lavelle C., Tanoto H., Wang Y., Chen K., Milton E., Zhou Y., Dong X. (2025). Minimally invasive healing of bone implant-cement interfaces by aerogel cement and remote heating. Device.

